# Particulate matter emissions during autopsies: a method to reduce exposure

**DOI:** 10.1007/s11356-022-20021-7

**Published:** 2022-04-14

**Authors:** Janis Dröge, Ibrahim El Moussaoui, Doris Klingelhöfer, Hannelore Held, David. A. Groneberg, Marcel A. Verhoff, Stefanie Plenzig

**Affiliations:** 1 Institute of Occupational, Social, and Environmental Medicine, Goethe University Frankfurt, Theodor-Stern-Kai 7, 60590 Frankfurt am Main, Germany; 2 Institute of Legal Medicine, Goethe University, University Hospital Frankfurt, Kennedyallee 104, 60596 Frankfurt am Main, Germany

**Keywords:** Autopsy, Bone dust, Face masks, Oscillating saws, Particle load reduction, Particulate matter, Particle size distribution

## Abstract

Particulate matter emitted during autopsies can serve as a vector for numerous viruses or bacteria and can lead to infections. Reducing the exposure of those particles in indoor working environments is, therefore, an important issue. To assess the health risk for employees in forensic medicine, we measure particulate matter in the ambient air during autopsies by using an aerosol spectrometer. The autopsies were performed with either an ordinary oscillating saw or an adapted saw with a suction unit. The particle emissions from both saws were compared to each other in order to evaluate whether a technical adaption leads to a particle reduction. Furthermore, the particle exposure reduction by wearing a face mask and variations in the background concentration in the room were analyzed. High particle concentrations were measured while using the ordinary saw. By using the adapted saw or wearing a face mask, the particle exposure could mostly be avoided. On the majority of the working days, an increase in the background concentration could be observed. Based on this knowledge, the use of a proper suction unit and wearing a face mask during autopsies is necessary. Besides, it is important to have sufficient ventilation in the room so that long-lasting high background concentrations can be prevented.

**Graphical abstract:**

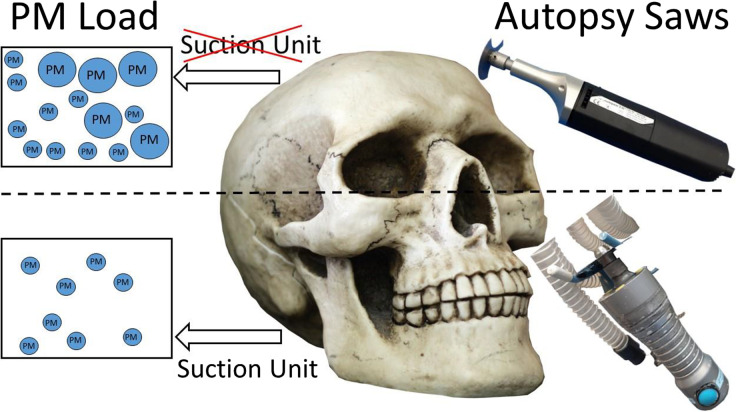

**Supplementary Information:**

The online version contains supplementary material available at 10.1007/s11356-022-20021-7.

## Introduction

Particulate matter (PM) consists of a complex mixture of solid and liquid particles in the air. These particles are emitted, for example, during combustion or abrasion processes. Particle formation, which takes place through chemical reactions of emitted precursor substances, also contributes to the total concentration (EPA 2020). PM consists of particles with a wide range of aerodynamic diameters. In monitoring air quality, mostly the categories PM_10_ (particulate matter with an aerodynamic diameter less than 10 µm), PM_2.5_ (particulate matter with an aerodynamic diameter less than 2.5 µm), PM_1_ (particulate matter with an aerodynamic diameter less than 1 µm), and PM_coarse_ (particles with diameters between 2.5 and 10 µm) are used (Air Quality Expert Group 2005).

High exposure to PM has numerous negative influences on human health and is therefore a major and current research field in medicine and environmental sciences. Even short-term exposures with high concentrations have a harmful potential and can lead to an increase in cardiovascular and respiratory mortality (Katsouyanni et al. 1997; Delfino et al. 1998; Orellano et al. 2020). Likewise, disorders in blood coagulation and an increase in the incidence of strokes can result from short-term exposure to high concentrations of PM (Wei et al. 2019; Matsuo et al. 2016). Under long-term exposure, the consequences can be even worse. Especially chronic respiratory diseases like chronic obstructive pulmonary disease (COPD), asthma, or cancer can be linked to chronic exposure to high concentrations of PM (Han et al. 2020; Yorifuji and Kashima 2019). Depending on their size, there are major differences in where they deposit in the respiratory tract. Whereas coarse particles tend to deposit in the upper airways, small particles can reach the area of the gas exchange and have the potential to act systemically (Brown et al. 2013; Darquenne 2012). According to an EU guideline, there is a limit value for the daily mean PM_10_ concentration of 50 µg/m^3^, which must not be exceeded on more than 35 days per year (Directive 2008/50/EC 2008).

Because people in Central Europe spend 80–90% of the day indoors (Federal Ministry for Environment, Nature Conservation and Nuclear Safety 2020), the composition of the ambient air in the rooms is a crucial factor for the total daily PM uptake. High concentrations of PM can last for hours due to the limited volume and the reduced ventilation. The indoor PM concentration is strongly affected by indoor sources (Meier et al. 2015). Depending on the occupation and the working environment, the PM uptake differs significantly. Employees in forensic medicine are one of these occupational groups with a notable risk of being exposed to a high amount of PM (Kernbach-Wighton et al. 1996, 1998; Pluim et al. 2018).

Autopsies are required, for example, when there is evidence of a nonnatural cause of death. For proper execution of the autopsy, the opening of the cranium is indispensable (Federal Office of Justice 2021). Therefore, commonly oscillating saws are used. The usage of these saws leads to high emission of potentially respirable material during the sawing process, which is scattered over several meters (Kernbach-Wighton et al. 1996; Wenner et al. 2017; Jones and Brosseau 2015; Noble et al. 1963). This material consists of bone dust, liquid aerosol, large droplets of blood, and cerebrospinal fluid with a high percentage of PM in its particle size distribution (Green and Yoshida 1990). In contrast to PM from natural sources or from burning fossil fuels, PM emitted during autopsies has further negative effects on human health. For instance, this kind of material can serve as a vector for numerous different biological hazards, like bacteria or viruses and other microbes. The morphology of these particles is very variable. Especially serrated particles have a high ability to adhere to mucous membranes and favor infections (Kernbach-Wighton et al. 1996). It has already been proven that there occurs an entry of tubercle bacteria into the ambient air during autopsies (Templeton et al. 1995). First studies also indicate that corpses of deceased COVID-19 patients have to be considered potentially infective during autopsies (Plenzig et al. 2021). Likewise, the human papillomavirus has been transferred, while material was scattered during surgery (Barrett and Garber 2003). Besides infection, intoxications can also occur. For example, cyanide poisoning occurred while a deceased person with cyanide poisoning was opened for a forensic autopsy (Seyit et al. 2020).

The purpose of this study is to evaluate the health risk for employees in forensic medicine by monitoring PM emissions during forensic autopsies. It was analyzed whether reduction of PM emission could be achieved by a technical adaption of the oscillating saw. Furthermore, the influence of wearing a particle filtering face mask while using the saw was analyzed.

## Materials and methods

### Oscillating autopsy saws

**Fig. 1:**
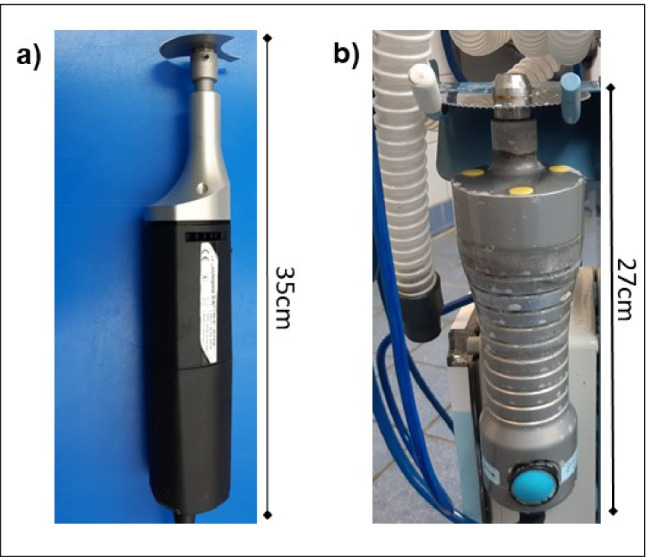
Oscillating saws used during the autopsies from **a** Bühler Instrumente (OS) and **b** Kugel Medical (AS)

In this study, two different types of oscillating autopsy saws were used and compared. The autopsy saw from Bühler Instrumente (article number 30.219.20) is an ordinary oscillating saw (OS) with a maximum of 24,000 oscillations per minute (Fig. 1a). The saw has no further equipment. The other autopsy saw used during the autopsies is from Kugel Medical (type SF-4000) with a maximum of 12,000 oscillations per minute (Fig. 1b). This saw is an adapted oscillating saw (AS) equipped with a suction unit to reduce the amount of material that is scattered in the surrounding area.

### Measurement of particulate matter

In this study, the PM concentration during autopsies was measured by an aerosol spectrometer of type *GRIMM 11-R*, which was connected to a laptop of the type Fujitsu Lifebook. Every 6 s, a value was generated for the PM fractions PM_10_, PM_2.5_, and PM_1_. The PM_coarse_ values are equal to the difference between PM_10_ and PM_2.5_. Besides a constant measurement of PM, temperature and humidity were monitored.

### Measurement protocol

The measuring interval was limited to the time interval around the opening of the cranium because of high variations in the duration of the autopsies. Measurement started 5 min before the saw was used to generate a proper value for the mean background concentration. The measurement continued for the whole time of using the saw and lasted for a further 5 min to maximize the amount of particles that moved in the direction of the spectrometer and finally gets analyzed.

The aerosol spectrometer was located 1.5 m away from the body in direction of the feet so that a realistic scenario for the exposure to the employees could be ensured.

Measurements took place at the Institute of Legal Medicine Frankfurt am Main in the period between May 31, 2016, and June 28, 2016. The measurements were carried out during normal autopsy time (9:00–16:30). The exact point of time when the autopsies took place was based on the circumstances of death and personnel availability. In total, 16 measurements were carried out and analyzed while the OS without the suction unit was used (OS 1–OS 16). Another 16 measurements were carried out and analyzed while the AS equipped with the suction unit was used (AS 1–AS 16). The dates and the point of time of the single measurements are given in Table 1.

**Table 1:** Dates and point of time for all autopsies performed with the OS and the AS

Date	Autopsies performed with the OS with the corresponding point of time	Autopsies performed with the AS with the corresponding point of time
May 31, 2016	OS 1 (9:30), OS2 (13:00)	
June 01, 2016	OS 3 (15:00)	
June 02, 2016	OS 4 (9:45), OS 5 (12:15), OS 6 (14:30)	
June 03, 2016	OS 7 (10:15), OS 8 (12:30)	
June 06, 2016	OS 9 (9:00), OS 10 (12:15)	
June 07, 2016	OS 11 (9:15), OS 12 (12:45)	AS 1 (15:00)
June 08, 2016		AS 2 (9:30)
June 09, 2016		AS 3 (10:00), AS 4 (12:00), AS 5 (15:00)
June 10, 2016		AS 6 (9:15), AS 7 (12:00)
June 13, 2016	OS 13 (10:15)	AS 8 (12:30)
June 15, 2016	OS 14 (16:00)	AS 9 (9:15), AS 10 (12:30)
June 16, 2016		AS 11 (9:45)
June 20, 2016		AS 12 (8:45)
June 21, 2016		AS 13 (9:30), AS 14 (13:30)
June 22, 2016		AS 15 (9:15)
June 27, 2016	OS 15 (12:15)	
June 28, 2016	OS 16 (12:45)	AS 16 (8:45)

The average sawing time when using the OS was 69 s. Due to the higher weight and a slightly restricted view during sawing, the sawing time is longer when using the AS. Here, an average working time of 141 s was necessary to completely open the cranium. The quality of the work is not affected by the suction unit.

**Fig. 2:**
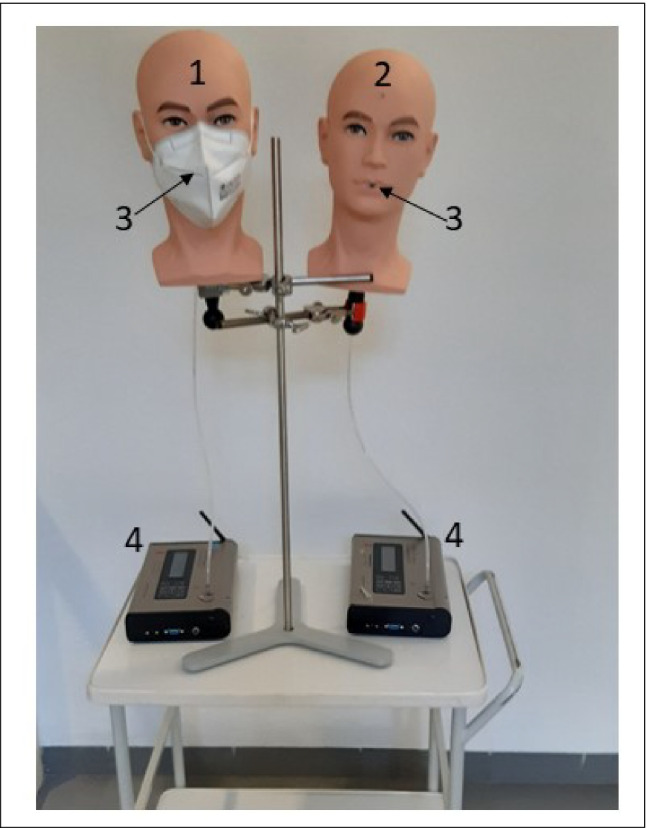
Dummy heads with face mask (**1**) and without face mask (**2**) with sample inlets (**3**) connected to the aerosol spectrometers (**4**)

In order to quantify the influence of particle filtering masks on particle exposure, another 5 measurements with the same protocol were carried out while the OS was in use. Here, measurements were performed simultaneously with two aerosol spectrometers of type GRIMM 11-R. The sample inlets were integrated into two dummy heads. One head was equipped with a face mask (FFP2 CE norm 2834) (Fig. 2).

### Statistics

The concentration of PM was measured separately for each autopsy. The background value (PM_bkgd_) was generated out of the mean concentration measured in a 5-min time interval before the saw was used. The PM value assigned to the sawing process (PM_saw_) was the mean PM concentrations measured during the sawing time itself and for another 5 min afterward. PM_bkgd_ and PM_saw_ were compared with a Wilcoxon signed-rank test. The measurements with the different saws are analyzed separately from each other.

For the comparison of the particle size distribution between PM_bkgd_ and PM_saw,_ the particles were grouped in the size categories of 2.5–10 µm, 1–2.5 µm, and < 1 µm. For the calculation of the range of particles with diameters between 2.5 and 10 µm, the concentration of PM_2.5_ was subtracted from the concentration of PM_10_. For the range of particles with a diameter between 1 and 2.5 µm, the concentration of PM_1_ was subtracted from the concentration of PM_2.5_.

The absolute concentration differences (PM_∆_) measured after the use of the saw were calculated by subtracting the value for PM_bkgd_ from that of PM_saw_. The concentration differences measured during the autopsies performed with the OS and those measured during the autopsies performed with the AS were compared to each other with a Mann–Whitney *U*-test. Particles of the fractions PM_coarse_, PM_10_, PM_2.5_, and PM_1_ were analyzed separately.

To determine the exposure reduction that can be achieved by wearing a face mask, the simultaneously measured values were compared with each other. In previous simultaneous test measurements without the use of a mask in the autopsy room during normal operation, an average deviation of 1.5% was found between the two aerosol spectrometers in the determination of the total particle mass. Due to this very small value, the deviation can be neglected when interpreting the values in the following measurements.

The single background concentrations, measured before each autopsy within one working day, were compared to each other in order to characterize shifts in the PM background level.

The statistical analyzes were performed using Graphpad Prism 9.

## Results

### Autopsies performed with the OS

The values for the PM background concentrations and the PM concentration while using the OS are given in Table 2.

**Table 2:** Minimum, maximum, and mean concentrations for different PM fractions measured for the background and while using the OS

	Particle size fraction	Min. [µg/m^3^]	Max. [µg/m^3^]	Mean [µg/m^3^]
PM_bkgd_	PM_coarse_	0.4	8.8	3.1
	PM_10_	4.8	47.8	23.3
	PM_2.5_	3.3	45.2	20.3
	PM_1_	2.6	43.5	19.1
PM_saw_	PM_coarse_	24.2	781.6	136.7
	PM_10_	55.9	952.3	185.3
	PM_2.5_	17.4	170.7	48.6
	PM_1_	7.0	53.2	26.0

Throughout the entire measurement period, maximum short-term concentrations (6-s values) of 11,735.4 µg/m^3^ for the PM_coarse_ fraction, 12,580.7 µg/m^3^ for the PM_10_ fraction, 845.3 µg/m^3^ for the PM_2.5_ fraction and 244.9 µg/m^3^ for the PM_1_ fraction were measured while using the OS.

**Fig. 3:**
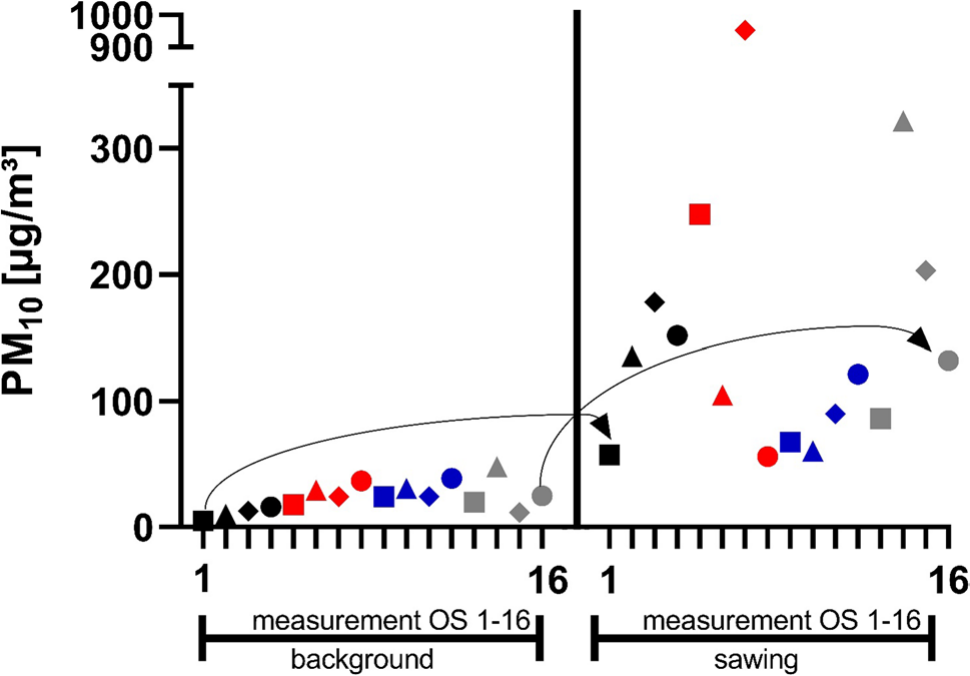
Comparison of the PM_bkgd_ values for the PM_10_ fraction and the corresponding values for PM_saw_ for all 16 autopsies performed (different symbol for each autopsy) with the OS (OS 1–16)

The concentration differences (PM_Δ_) between PM_bkgd_ and PM_saw_ for every single autopsy carried out with the OS are given in Table 3. Figure 3 shows the PM_bkgd_ values and the corresponding values for PM_saw_ for the PM_10_ fraction for all 16 measurements.

**Table 3:** Concentration differences (PM_Δ_) between PM_bkgd_ and PM_saw_ for all autopsies performed with the OS

Autopsy	PM_coarse_ [µg/m^3^]	PM_10_ [µg/m^3^]	PM_2.5_ [µg/m^3^]	PM_1_ [µg/m^3^]	Autopsy	PM_coarse_ [µg/m^3^]	PM_10_ [µg/m^3^]	PM_2.5_ [µg/m^3^]	PM_1_ [ug/m^3^]
OS 1	38.3	52.4	14.1	4.4	OS 9	30.7	43.4	12.7	4.0
OS 2	81.9	125.6	43.7	28.2	OS 10	22.5	29.7	7.1	2.9
OS 3	142.5	165.2	22.6	3.6	OS 11	45.6	65.6	20.0	6.2
OS 4	118.4	135.8	17.3	3.1	OS 12	76.0	82.6	6.6	− 2.0
OS 5	175.4	230.2	54.8	14.4	OS 13	59.7	66.0	6.3	0.8
OS 6	67.9	75.5	7.6	− 1.6	OS 14	243.2	273.9	30.7	− 3.8
OS 7	772.8	928.1	155.3	39.7	OS 15	157.8	191.5	33.7	7.4
OS 8	17.9	19.2	1.3	− 0.1	OS 16	87.3	107.2	19.8	4.2

A comparison of the concentrations for PM_bkgd_ and PM_saw_ shows that sawing with the OS leads to PM concentration increases for the fractions PM_coarse_, PM_10_, and PM_2.5_. Also, for the PM_1_ fraction, increases were measured for most autopsies. However, there was a slight decrease in the measured values during three autopsies.

The Wilcoxon signed-rank test shows for all fractions significant differences between PM_bkgd_ and PM_saw_ (Table 4).

**Table 4:** Comparison of the PM_bkgd_ and the PM_saw_ values for the autopsies performed with the OS for different particle size fractions

Particle size fraction	*P*-value
PM_coarse_	< 0.0001
PM_10_	< 0.0001
PM_2.5_	< 0.0001
PM_1_	0.0052

Significance level was set at *p* < 0.05. Statistical data based on the Wilcoxon signed-rank test

**Fig. 4:**
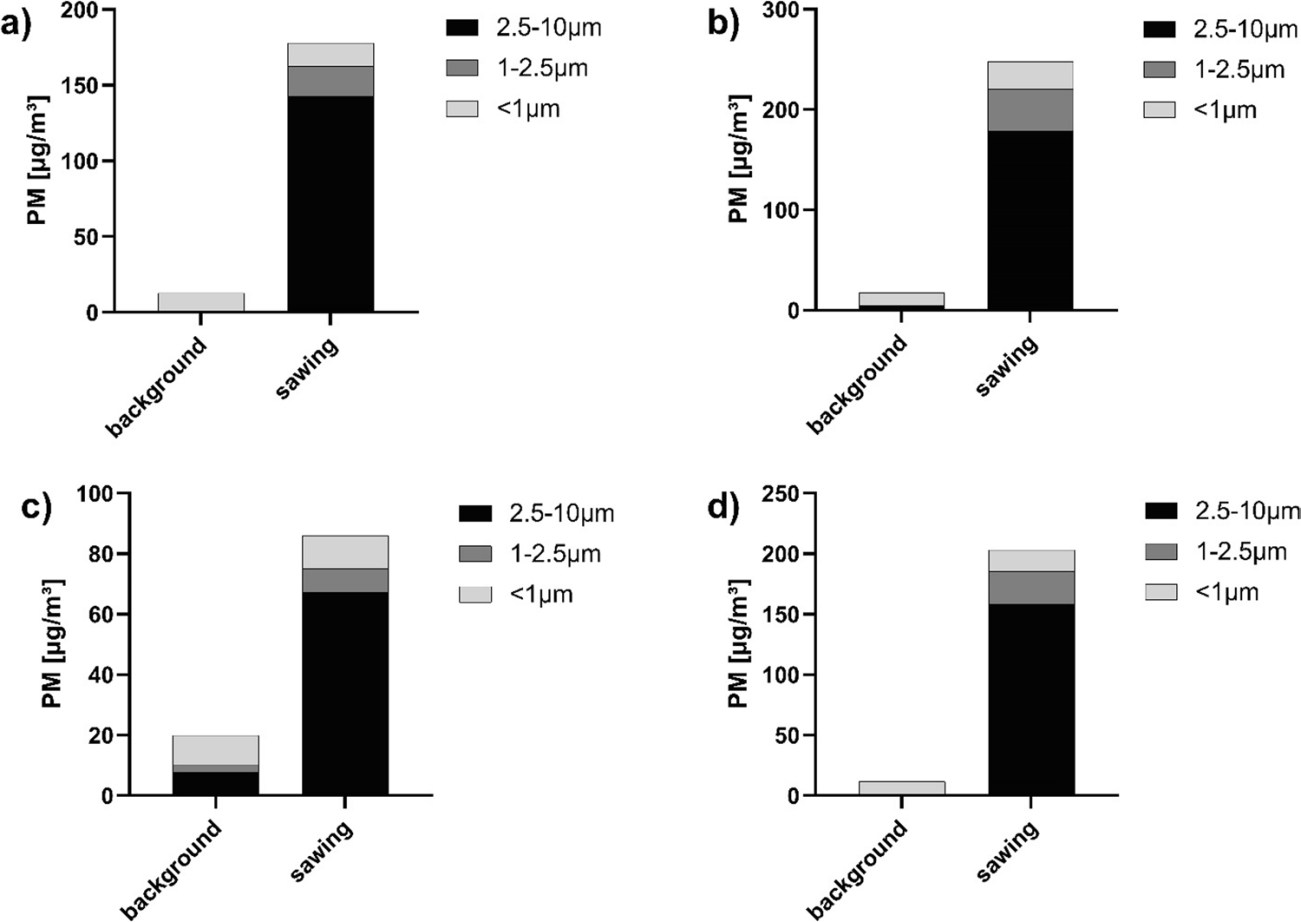
Examples of the particle concentrations and particle size distributions (background and sawing) measured during different autopsies performed with the OS for **a** OS 3, **b** OS 5, **c** OS 13, and **d** OS 15

There were significant differences in the increase in particle concentration and particle size distribution between the single autopsies measured (Fig. 4).

The coarse particles always show the highest concentration increases. For that reason, there is a shift of the particle size distribution in favor of the coarse particles in all measurements.

### Autopsies performed with the AS

The values for the PM background concentrations and the PM concentration while using the AS are given in Table 5.

**Table 5:** Minimum, maximum, and mean concentrations for different PM fractions measured as background and while using the AS

	Particle size fraction	Min. [µg/m^3^]	Max. [µg/m^3^]	Mean [µg/m^3^]
PM_bkgd_	PM_coarse_	0.6	11.2	3.8
	PM_10_	9.3	54.6	24.1
	PM_2.5_	8.4	47.0	20.2
	PM_1_	7.7	44.4	19.1
PM_saw_	PM_coarse_	2.1	29.0	5.3
	PM_10_	11.3	52.1	25.1
	PM_2.5_	8.8	44.5	19.8
	PM_1_	7.6	41.6	18.0

Throughout the entire measurement period, maximum short-term concentrations (6-s values) of 61.2 µg/m^3^ for the PM_coarse_ fraction, 85.2 µg/m^3^ for the PM_10_ fraction, 59.9 µg/m^3^ for the PM_2.5_ fraction, and 55.3 µg/m^3^ for the PM_1_ fraction were measured while using the AS.

**Fig. 5:**
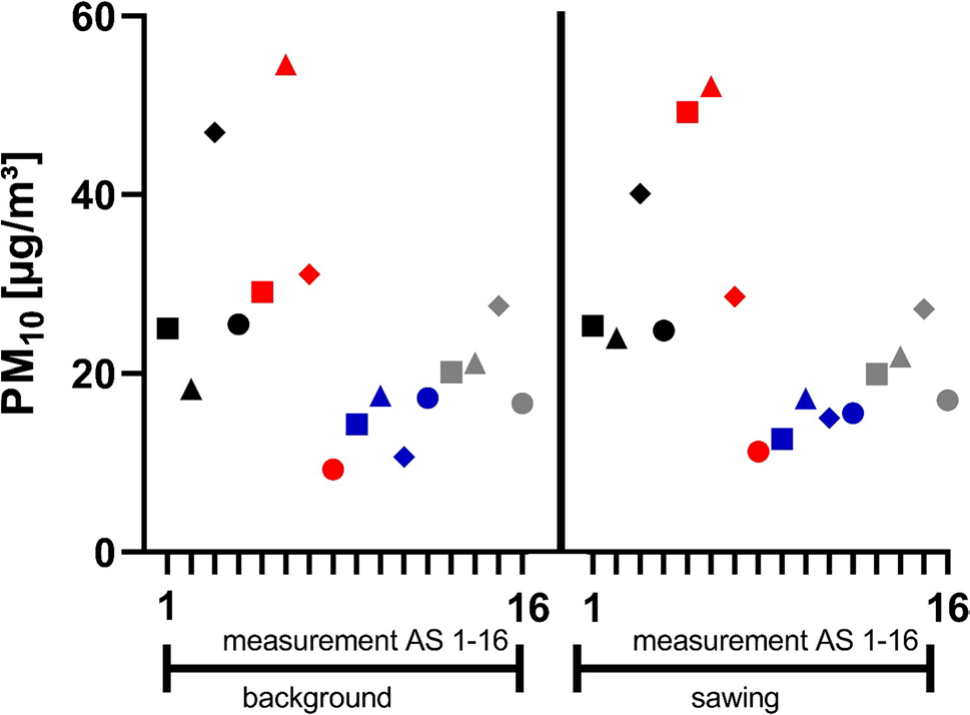
Comparison of the PM_bkgd_ values for the PM_10_ fraction and the corresponding values for PM_saw_ for all 16 autopsies (different symbol for each autopsy) performed with the AS (AS 1–16)

The concentration differences (PM_Δ_) between PM_bkgd_ and PM_saw_ for every single autopsy carried out with the AS are given in Table 6. Figure 5 shows the PM_bkgd_ values for the PM_10_ fraction and the corresponding values for PM_saw_ for all 16 measurements.

**Table 6:** Concentration differences (PM_Δ_) between PM_bkgd_ and PM_saw_ for all autopsies performed with the AS

Autopsy	PM_coarse_ [µg/m^3^]	PM_10_ [µg/m^3^]	PM_2.5_ [µg/m^3^]	PM_1_ [µg/m^3^]	Autopsy	PM_coarse_ [µg/m^3^]	PM_10_ [µg/m^3^]	PM_2.5_ [µg/m^3^]	PM_1_ [µg/m^3^]
AS 1	0.9	0.3	− 0.6	− 1.0	AS 9	− 1.0	− 1.6	− 0.7	− 0.1
AS 2	1.5	5.7	4.2	3.5	AS 10	1.0	− 0.3	0.6	0.6
AS 3	1.1	− 6.9	− 8.0	− 7.9	AS 11	1.8	4.3	2.5	2.2
AS 4	− 1.1	− 0.7	0.3	0.5	AS 12	− 2.0	− 1.7	0.3	0.4
AS 5	17.9	20.1	2.3	0.1	AS 13	0.6	− 0.2	− 0.8	− 1.3
AS 6	0.0	− 2.5	− 2.5	− 2.8	AS 14	1.6	0.7	− 0.9	− 0.6
AS 7	− 0.3	− 2.5	− 2.2	− 1.0	AS 15	1.3	− 0.4	− 1.7	− 2.5
AS 8	1.5	2.0	0.5	0.3	AS 16	0.1	0.3	0.2	− 0.5

No trend can be observed when using the AS. From the time sawing begins, there are both concentration increases and decreases. The Wilcoxon signed-rank test shows no significant differences for all fractions between the background values and the corresponding values measured while the saw is used (Table 7).

**Table 7:** Comparison of the PM_bkgd_ and the PM_saw_ values for the autopsies performed with the AS for different particle size fractions

Particle size fraction	*P*-value
PM_coarse_	0.1297
PM10	0.9298
PM_2.5_	0.5966
PM_1_	0.3484

Significance level was set at *p* < 0.05. Statistical data based on the Wilcoxon signed-rank test

**Fig. 6:**
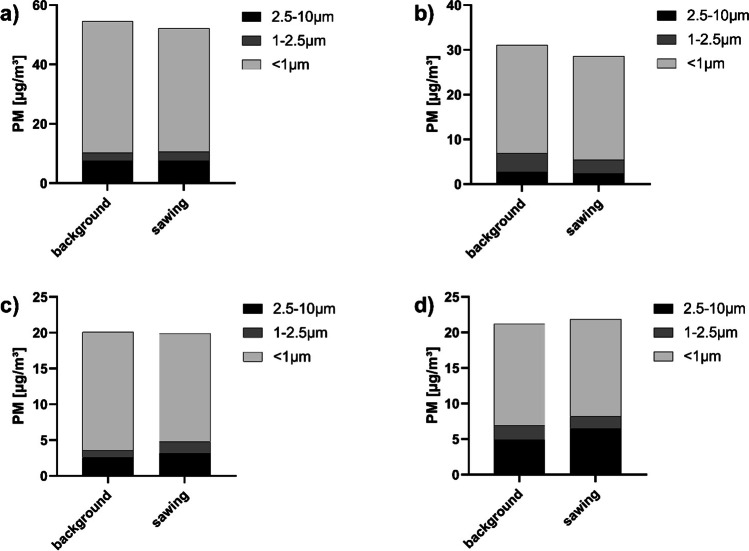
Examples of the particle concentrations and particle size distributions (background and sawing) measured during different autopsies performed with the AS for **a** AS 6, **b** AS 7, **c** AS 13, and **d** AS 14

The particle size-specific analysis also does not show a consistent pattern (Fig. 6).

Overall, the changes in the particle size spectrum are only marginal. There are slight shifts in favor of the finer particles as well as slight shifts in favor of the coarse particles.

### Comparison between the OS and the AS

**Fig. 7:**
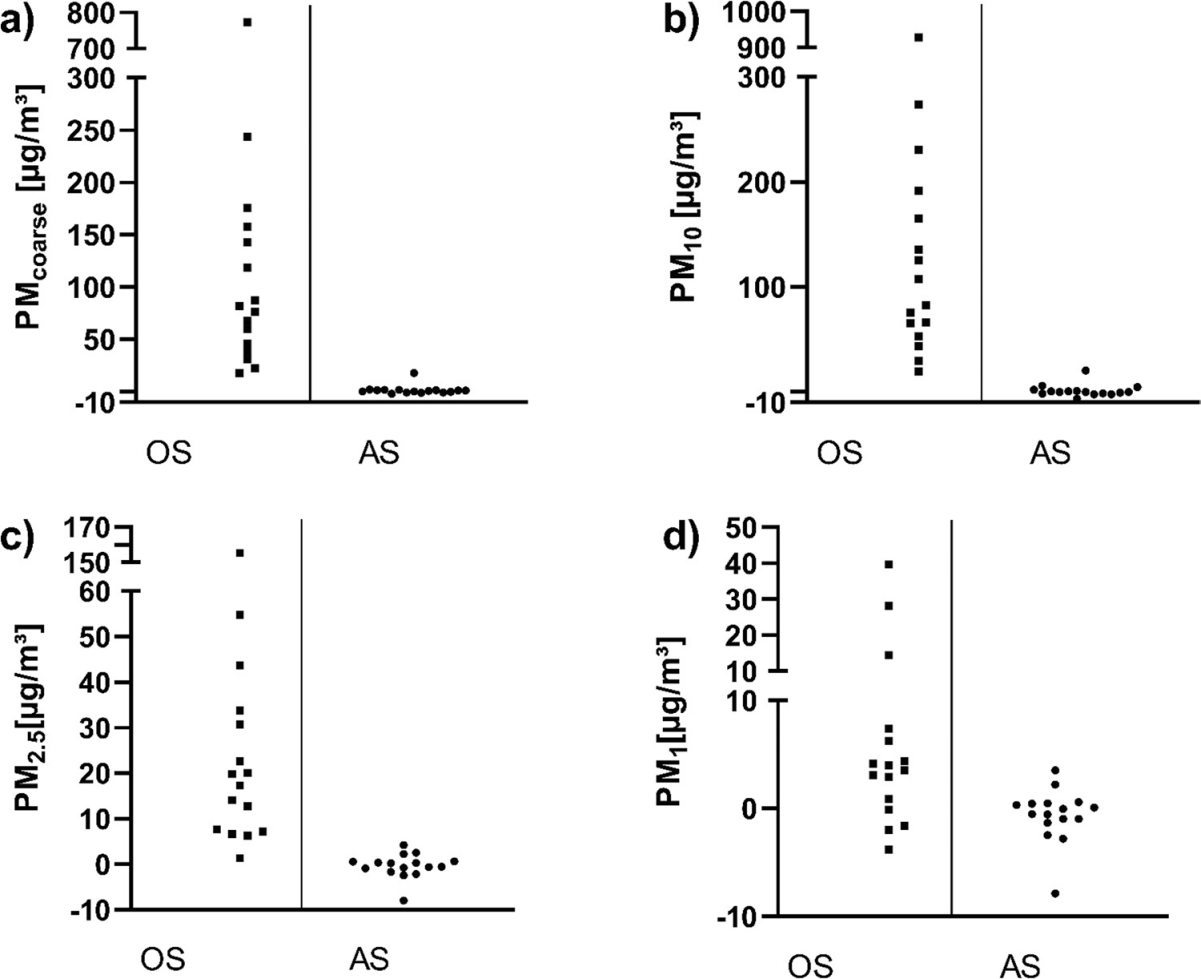
Comparison between the PM_Δ_ values from the autopsies performed with the OS and those performed with the AS

When comparing the PM_Δ_ values measured when working with the OS with those measured when working with the AS, significant differences can be observed (Fig. 7).

The Mann–Whitney *U*-test shows a significant difference for all particle size fractions (Table 8).

**Table 8:** Comparison of the PM_Δ_ values of the autopsies performed with the OS and those performed with the AS for different particle size fractions

Particle size fraction	*P*-value
PM_coarse_	< 0.0001
PM_10_	< 0.0001
PM_2.5_	< 0.0001
PM_1_	0.0039

Significance level was set at *p* < 0.05. Statistical data based on the Mann–Whitney *U*-test

### Influence of a face mask on the PM exposure

**Fig. 8:**
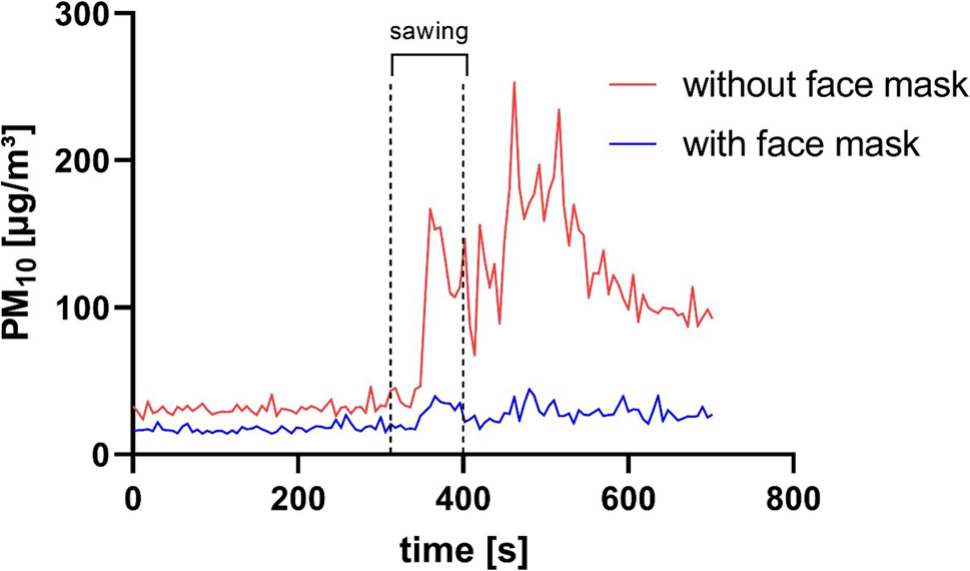
PM Concentration profile during an autopsy measured with and without a face mask in front of the sample inlet of an aerosol spectrometer

A comparison of PM concentration measured with and without a face mask in front of the sample inlet of an aerosol spectrometer shows significant differences (Fig. 8).

Before sawing begins, it is already apparent that a reduction in PM exposure can be achieved through the use of a mask. However, it is only after the saw has been used that the full potential of the protective effect becomes apparent. If a very high exposure occurs without the mask, the exposure level remains approximately constant at a low level when the mask is used. Table 9 shows the reduction of PM_saw_ by measuring behind a face mask.

**Table 9:** PM_saw_ reduction when measuring behind a face mask

	PM_saw_ reduction [%]		
	PM_10_ [µg/m^3^]	PM_2.5_ [µg/m^3^]	PM_1_ [µg/m^3^]
Autopsy 1	64.9	49.1	38.6
Autopsy 2	61.4	49.9	37.9
Autopsy 3	66.1	52.6	46.7
Autopsy 4	76.6	60.8	44.3
Autopsy 5	73.1	55.5	42.1

For all particle fractions, a significant reduction in particle concentration can be achieved if measurements were performed behind a face mask for each individual autopsy.

### Shifts in the background concentration during the working days

**Fig. 9:**
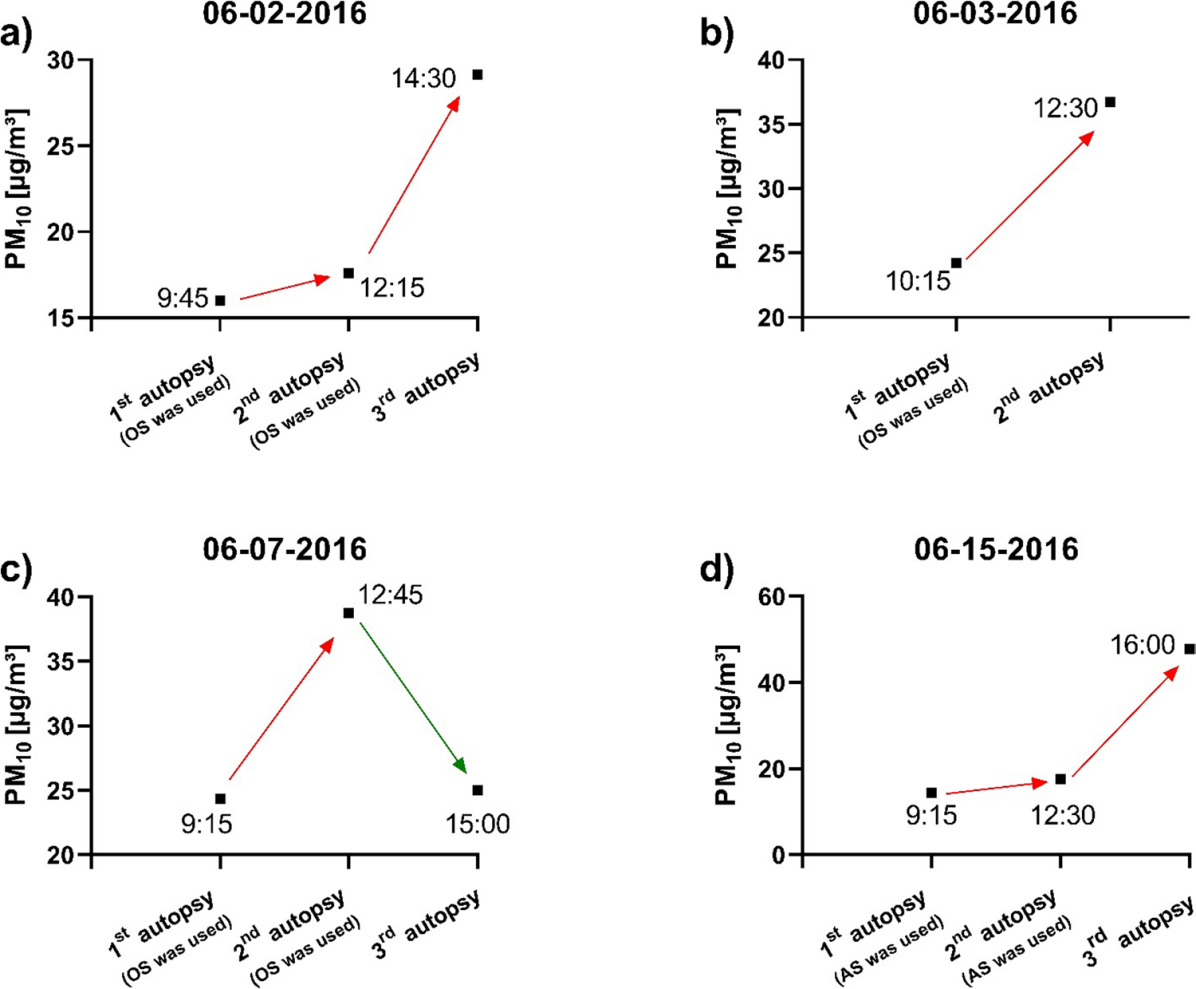
Examples for changes in the background concentrations for PM_10_ during a working day measured on **a** June 2, 2016, **b** June 3, 2016, **c** June 7, 2016, and **d** June 15, 2016. Red arrows: concentration increases, green arrows: concentration decreases

Both increases and decreases can be observed when analyzing the shifts in the background concentration. Figure 9 shows background concentration profiles for four different working days with their characteristic shifts.

The PM_bkgd_ trends for June 2, 2016, and June 3, 2016 show increases in the background concentration within the working days. On both days, solitary the OS was used.

On June 7, 2016, despite high emissions during the sawing processes (1st and 2nd autopsy), there was a drop in the background concentration later in the day.

Although no increase in concentration was measured during sawing with the AS on June 15, 2016 (1st and 2nd autopsy), a slight increase in the background concentration could be observed.

Overall, over the entire measurement period, rather increases in the background concentration during the working days could be observed.

### Temperature and relative humidity

The measured values for temperature and relative humidity during the autopsies on single working days are given in Table 10.

**Table 10:** Measured values for temperature and relative humidity during the autopsies on the single working days

Date	Measurements	Temperature [°C]	Rel. humidity [%]
May 31, 2016	OS 1, OS 2	23.7–24.2	46.1–47.6
June 1, 2016	OS 3	23.8	55.0
June 2, 2016	OS 4, OS 5, OS 6	23.1–23.9	55.6–56.2
June 3, 2016	OS 7, OS 8	23.8–25.5	50.5–53.8
June 6, 2016	OS 9, OS 10	23.5–23.5	49.3–54.2
June 7, 2016	OS 11, OS 12, AS 1	23.1–24.1	45.8–50.3
June 8, 2016	AS 2	23.6	48.2
June 9, 2016	AS 3, AS 4, AS 5	23.0–23.9	43.6–46.1
June 10, 2016	AS 6, AS 7	22.7–23.9	45.9–46.2
June 13, 2016	OS 13, AS 8	24.1–25.4	52.6–53.7
June 15, 2016	AS 9, AS 10, OS 14	23.9–24.4	52.9–53.0
June 16, 2016	AS 11	22.5	49.3
June 20, 2016	AS 12	22.9	46.7
June 21, 2016	MS 13, MS 14	22.5–23.3	56.0–58.3
June 22, 2016	AS 15	23.3	58.7
June 27, 2016	OS 15	22.9	49.9
June 28, 2016	OS 16, AS 16	23.4–24.2	53.8–54.0

There were minor fluctuations in temperature and relative humidity within the different working days.

## Discussion

### PM concentrations measured during the autopsies

Oscillating autopsy saws lead to high emission of potentially respirable material during the sawing process (Kernbach-Wighton et al. 1996). Because this material can serve as a vector for many different biological hazards or toxic substances, it can cause infection or poisoning when inhaled. Within the framework of the study conducted, these emissions were characterized.

It could be observed that the particle concentration increased significantly during an autopsy when sawing with the OS. The increase in the whole particle concentration is mainly due to an increase in the concentration of the coarse particles. The particle concentration of the medium and finer fraction increased only slightly. In some exceptional cases, there was even a slight decrease in the finest particle fraction. This can be explained by an increase in collision processes between fine and coarse particles at a higher number of coarse particles in total. These collisions result in further growth of the coarse particles (Zarzycki et al. 2020).

In terms of absolute particle concentration after sawing, large differences could be observed.

The measurements were carried out under real conditions so that different sawing times and cutting patterns were necessary depending on the condition of the human body. The bodies can also show differences in the degree of moisture. This can have an overall effect on the amount of material that is emitted into the ambient air during the sawing process (Pluim et al. 2018). Furthermore, under real working conditions, it cannot be ruled out that the air circulation in the autopsy room changes. Rapid movements of the employees or a slight air draft can already cause the emitted particles to spread to the opposite side of the spectrometer.

Generally, no significant increase in the particle concentration could be observed for any fraction when the AS with the suction unit was used. In the direct comparison of both saws, the very high emission of coarse particles, in particular, did not occur while using the AS. The suction unit is therefore suitable for preventing an increase in the total particle load in the ambient air, especially for the coarse particles, while working with an oscillating saw. Since the AS was normally operated at a lower oscillation frequency than the OS, it can be assumed that an additional particle reduction was thus achieved (Pluim et al. 2018).

Concentration increases in isolated autopsies are probably due to the inability of the suction device to sufficiently eliminate particles at certain cutting angles or cutting speeds.

The measurements with the particle filtering mask show that face masks can lead to a significant reduction in PM exposure.

### Background concentration

When observing the changes in the background concentration in the autopsy room during one day, no clear trend could be identified. Despite very high emissions during the autopsies, in many cases, the background value increased only slightly during the day. For example, when on June 3, 2016, the first autopsy was performed with the OS, an enormous increase of 928.1 µg/m^3^ was detected for the fraction PM_10_ after the saw was used. Two hours later, only a value of 36.7 µg/m^3^ was measured as background before the following autopsy began. This is only a concentration increase of 12.5 µg/m^3^ compared to the background level earlier that day. On other working days, there was even a slight drop in background concentration despite high emissions during the sawing process.

Occasionally, increasing background values could be observed despite the lack of emissions during autopsies with the suction unit. Different reasons might be crucial factors to influence variations in the background concentration during a day. For example, changes in relative humidity may affect particle concentration. An increase in the relative humidity in indoor air leads to a significant elimination of the particles. On the other side, decreases in the relative humidity can lead to a high burden of PM in the ambient air (Kraus and Šenitková 2017). During one working day, both increases and decreases in relative humidity occurred. The influence of the temperature on the PM indoor concentration is rather low (Kraus and Šenitková 2017). In addition, coarse particles normally are deposited from the ambient air within hours by sedimentation processes (Qian et al. 2008). In the present study, in particular, these kinds of particles were emitted during sawing. Fine particles can also be eliminated from the ambient air through collision processes (Zarzycki et al. 2020). Changes in the ventilation of the room can also influence the particle distribution and thus the concentration at the autopsy table throughout the day (Fromme et al. 2007).

Overall, the background concentration seems to depend on a complex interaction of particle input in the ambient air as well as sedimentation, variations in humidity, and air circulation in the autopsy room. Considering all working days studied, increases rather than decreases in concentration could be observed. In addition to particle reduction during sawing itself, regular ventilation of the autopsy room is necessary to reduce the accumulation processes.

### Medical risk assessment

During the sawing process, mainly particles were generated, which can be assigned to the coarse fraction. However, finer particles were also emitted into the ambient air. In medical risk assessment, the initial focus is on the finer particle fraction. These particles have such a small aerodynamic diameter that they can reach the alveoli region and act systemically. The coarse particles that are mainly formed during the sawing process do not have the ability to reach the alveoli region. They usually deposit in the upper respiratory tract and can be eliminated by the mucociliary system (Brown et al. 2013; Darquenne 2012). In principle, their systemically harmful potential is significantly lower than that of the finer particles.

In the case of an autopsy, it must be considered that the particles emitted have the potential to promote infections and intoxications if they come into contact with the mucous membranes (Kernbach-Wighton et al. 1996).

There are no definitive indoor limit values for PM. However, a guideline of the World Health Organization (WHO) assumes that there are no differences in the harmful potential between PM from indoor sources and PM from outdoor sources, so the limit values can be adopted (WHO Regional Office for Europe 2010).

Of the 16 PM_saw_ values measured during the autopsies performed with the OS, 12 exceeded the limit value of 50 µg/m^3^. From the autopsies with the AS, only 2 values exceeded the limit value. Even if values in this magnitude decrease relatively quickly, it must be assumed that on very busy days with low air exchange, exceedances of the limit values can also occur as a daily average. The highest PM value measured during an autopsy was 12,581 µg/m^3^. Although these high PM concentrations only last for a short period of time, they can have a significant impact on human health (Katsouyanni et al. 1997; Delfino et al. 1998).

Overall, it can be assumed that employees in forensic medicine are exposed to a high particle concentration when using an OS. The use of a suitable suction unit can significantly reduce this risk.

In former studies within a controlled environment, it has already been proven that a lower sawing frequency and a higher contact load lead to lower particle concentrations (Pluim et al. 2018). The use of manual saws or an oscillating saw with an integrated spray tube that runs on water can also reduce the emission of PM (Kernbach-Wighton et al. 1996; Wenner et al. 2017). Furthermore, a particle reduction can be achieved by using a table with a built-in ventilation system (Orellano et al. 2020; Pluim et al. 2018). For an adapted saw with a suction unit, a significant reduction in particle number concentration was observed for particles < 5 µm (Kernbach-Wighton et al. 1998). Numerous methods are therefore available to significantly reduce the particle concentration at the autopsy table. If the use of an oscillating saw without a suction unit or other additional protective mechanisms is unavoidable, it is advisable to wear medical protective equipment consisting of a particle filtering mask and safety goggles. In particular, the effectiveness of a face mask was demonstrated in this study. In addition to adjusting the saw itself or wearing protective equipment, attention should be paid to adequate ventilation of the autopsy room.

## Conclusion

In this study, it could be demonstrated under real conditions that ordinary oscillating saws used in forensic autopsies have the potential to cause high emissions of PM. Short-term values for the PM_10_ fraction of 12,581 µg/m^3^ were measured. The average PM values during the sawing process reached values up to 952.3 µg/m^3^ for the PM_10_ fraction. PM concentrations of this magnitude have a high potential to harm human health. The emitted particles mainly belonged to the coarse fraction. Although these coarse particles emitted during autopsies cannot reach the deeper respiratory tract and are largely eliminated by the mucociliary system, they can still lead to infections or intoxications.

While working with the AS, it could be observed that the suction unit reliably prevented the increase in particle concentration. However, careful handling is a prerequisite for this. Even if the work with the AS is associated with a slightly increased expenditure of time, this should not be dispensed with. Wearing particle filtering masks is another effective measure that can significantly reduce PM exposure.

It was shown that there were increases in the background concentration during the majority of the working days. Thus, in addition to the use of a suction unit, sufficient ventilation of the room is necessary.

If a technical adaptation of the saw, for example, with a suction unit, is not available, it is even more important to pay attention to sufficient protective equipment.

## Supplementary Information

Below is the link to the electronic supplementary material.

Supplementary file1 (XLSX 15 kb)

Supplementary file2 (XLSX 303 kb)

Supplementary file3 (XLSX 1409 kb)

Supplementary file4 (XLSX 264 kb)

## Data Availability

All data generated or analyzed during this study are included in the supplementary material of this article.
